# Expression of glycosylated human prolactin in HEK293 cells and related N-glycan composition analysis

**DOI:** 10.1186/s13568-019-0856-8

**Published:** 2019-08-29

**Authors:** Felipe D. Silva, João E. Oliveira, Renan P. Freire, Miriam F. Suzuki, Carlos R. Soares, Paolo Bartolini

**Affiliations:** Biotechnology Center, Instituto de Pesquisas Energéticas e Nucleares, IPEN, CNEN/SP, Avenida Prof. Lineu Prestes, 2242, Cidade Universitária, São Paulo, 05508-000 Brazil

**Keywords:** N-Glycoprofiling, N-Glycans, Human prolactin, HEK293 cell, MALDI–TOF-MS

## Abstract

Prolactin (PRL) is a hormone produced by the pituitary gland with innumerable functions, such as lactation, reproduction, osmotic and immune regulation. The present work describes the synthesis of hPRL in human embryonic kidney (HEK293) cells, transiently transfected with the pcDNA-3.4-TOPO^®^ vector carrying the hPRL cDNA. A concentration of ~ 20 mg/L, including glycosylated (G-hPRL) and non-glycosylated (NG-hPRL) human prolactin, was obtained, with ~ 19% of G-hPRL, which is higher than that observed in CHO-derived hPRL (~ 10%) and falling within the wide range of 5–30% reported for pituitary-derived hPRL. N-Glycoprofiling analysis of G-hPRL provided: (i) identification of each N-glycan structure and relative intensity; (ii) average N-glycan mass; (iii) molecular mass of the whole glycoprotein and relative carbohydrate mass fraction; (iv) mass fraction of each monosaccharide. The data obtained were compared to pituitary- and CHO-derived G-hPRL. The whole MM of HEK-derived G-hPRL, determined via MALDI–TOF-MS, was 25,123 Da, which is 0.88% higher than pit- and 0.61% higher than CHO-derived G-hPRL. The main difference with the latter was due to sialylation, which was ~ sevenfold lower, but slightly higher than that observed in native G-hPRL. The “in vitro” bioactivity of HEK-G-hPRL was ~ fourfold lower than that of native G-hPRL, with which it had in common also the number of N-glycan structures.

## Introduction

Human prolactin (hPRL) is a pituitary hormone, which has an essential role on mammopoiesis. It also exerts many other physiological functions on behavior and brain and on immune responses, metabolism and electrolyte balance (Bernichtein et al. 2010; Capone et al. 2015; Goffin et al. 2002). This hormone has been defined as the “regulator of maternal behavior” (Sinha 1995), but more recent studies started to consider it also as a “regulator of paternal behavior” (Gettler et al. 2012). Its clinical importance, especially for diagnostic purposes, is related to lactation problems and infertility in women but also to the fact that elevated circulating or locally produced hPRL levels are associated with the risk of breast and prostate cancer (Fernandez et al. 2010; Suzuki et al. 2012; Tworoger et al. 2004).

hPRL is a 199 amino acid-polypeptide, whose theoretical molecular mass (MM) of 22,897.75 Da, calculated from the amino acid sequence (Wu et al. 2003), has been perfectly confirmed by our research group via MALDI–TOF-MS on different CHO-, C127-, *E. coli*- and pituitary-derived preparations. This provided an average value of 22,910.3 Da, which is only 0.055% higher than the theoretical one (Capone et al. 2015; Heller et al. 2010; Soares et al. 2006; Wu et al. 2003).

This protein has a single potential N-glycosylation site located at Asn-31, which is partially occupied (5–30%) in the pituitary or in the recombinant forms of the hormone, providing MM values of 24,903 Da, 24,970 Da and 25,139 Da for pituitary-, CHO- and C127-derived G-hPRL, respectively (Capone et al. 2015). We are dealing therefore with one of the simplest types of glycosylation macroheterogeneity: one protein population with one, and one without a single N-linked glycan. This allowed us to determine, exclusively with basis on glycoprofiling analysis, (i) the monosaccharide composition of each N-glycan and of the entire glycoprotein; (ii) the average N-glycan mass; (iii) the whole glycoprotein mass and, consequently, (iv) the percent MM exclusively due to the carbohydrate moiety (Capone et al. 2015). After validation of this methodology, it has been possible, in previous work, just by adding MALDI–TOF-MS determination, to obtain the same parameters together with the average glycosylation site-occupancy, in more complex poly-glycosylated proteins (Ribela et al. 2017; Sant’Ana et al. 2018).

G-hPRL has been indeed considered the major post-translational modification of NG-hPRL, the two forms being co-secreted from childhood to the end of puberty, but the physiological significance of G-hPRL is still not well elucidated (Fideleff et al. 2012; Freeman et al. 2000). It has been observed, moreover, that G-hPRL has an approximately fourfold lower potency, compared to NG-hPRL, with reduced lactotrophic and mitogenic activity (Heller et al. 2010; Price et al. 1995; Shelikoff et al. 1994; Sinha 1995).

The present study gives continuity to our previous works that analyzed the influence of the host cell on G-hPRL carbohydrate structures and composition and on its biological activity (Capone et al. 2015; Heller et al. 2010; Soares et al. 2006). Recombinant G-hPRL has been synthesized in two specific strains of human embryonic kidney cells (HEK-293T and HEK293F), comparing all determined parameters with those obtained in native pituitary- and in CHO-derived G-hPRL. It is important to emphasize that while recombinant hPRL, and recombinant pituitary hormones in general, are always widely synthesized in different types of host cells and largely applied to human diagnosis and therapy, the comparison between their structures and bioactivities with the natural forms of these proteins is almost always neglected.

## Materials and methods

### Human cells line HEK293

For this work, the adherent HEK293T (ATCC^®^ CRL-11268™) (Sant’Ana et al. 2018) and HEK293F™ suspension cell line (Life Technologies, Carlsbad, CA, USA), were used for the Expi293^®^ Expression System Kit (Life Technologies, Carlsbad, CA, USA), following manufacturer’s instructions.

### Culture conditions and transfection of HEK293T adherent cells

HEK293T cells were maintained in incubator at 37 °C, 5% CO_2_. They were transfected with pEDdc-hPRL and p658-hPRL vectors described in previous work (Soares et al. 2000) using Lipofectamine™ and following the protocol described by Sant’Ana et al. (2018). Cells were cultured in 10 cm^2^ petri dishes with RPMI 1640 medium supplemented with 10% fetal bovine serum (FBS) until reaching 98% confluence. Sixteen hours after transient transfection, the medium was changed to serum free CHO-S-SFM II medium (Invitrogen, Carlsbad, CA, USA). During 4 days, 100% of the medium was collected and replaced every 2 days, being stored at − 80 °C. Samples were analyzed by SDS-PAGE and RP-HPLC. The Xfect^®^ polymer transfection method (Clontech, Mountain View, CA, USA) was applied to HEK293T cells, cultured as previously described (Sant’Ana et al. 2018). Cell transfection was carried out with 30 μg of pcDNA 3.4-TOPO-hPRL vector, dissolved in 600 μL of Xfect™ Reaction buffer, after vortexing for 5 s as recommended. Xfect™ Polymer (9 μL) was added to the DNA, vortexing again for 10 s. The reaction was incubated for 10 min at room temperature to form the nanoparticles (polymer-DNA complex). Cells had the media changed to 10 mL Expi293™ Expression Medium, without FBS and with penicillin (50 units/mL) and streptomycin (50 μg/mL), adding then 600 μL of the polymer-DNA complex. The transfected cells were incubated for 16–20 h and the medium was discarded once and renewed with 10 mL Expi293™ Expression Medium, supplemented with 50 μL Enhancer 1 and 500 μL Enhancer 2 from the ExpiFectamine™ 293 Transfection kit. The conditioned medium was collected every 2 days, for a total of 4 days of production. Conditioned medium and corresponding 1 mL aliquots were stored at − 80 °C.

### Culture conditions and transfection of HEK293F suspension cells

pcDNA™ 3.4-TOPO^®^ (30 μg) was used to transfect 7.5 × 10^7^ EXPI293F™ suspension cells (2.5 × 10^6^ cells/mL in 30 mL) in a 125 mL erlenmeyer, using 81 µL of ExpiFectamine™ transfectant agent. After 16 h of reaction, 150 µL of Enhancer 1 and 1.5 mL of Enhancer 2 were added and the culture was maintained in incubator at 37 °C, 8% CO_2_, in orbital shaker at 125 rpm. One milliliter of conditioned media was collected each day during 4 days and stored at − 80 °C, being analyzed by SDS-PAGE and RP-HPLC.

### Sodium dodecyl sulfate polyacrylamide gel electrophoresis (SDS-PAGE) and Western blotting

Conditioned media containing recombinant hPRL were analyzed on 15% SDS-PAGE under non-reducing conditions (Soares et al. 2000). Coomassie Brilliant Blue G-250 was used for the staining. For Western blotting, the semi-dry transfer technique was used on a nitrocellulose membrane, with anti-hPRL polyclonal antiserum produced in rabbit (1:5000) and goat anti-rabbit IgG conjugated to horseradish peroxidase (1:10,000). The anti-hPRL antiserum, NIDDK-anti-hPRL-3 (AFP-C11580), obtained by Dr. A. F. Parlow from the National Hormone and Pituitary Program (Torrance, CA, USA) and the anti-rabbit IgG secondary antibody produced in goat and conjugated to horsehadish peroxidase (HRP) (Signalway Antibody LLC, College Park, MA, USA) were used. Protein visualization was performed with Luminata Forte Western HRP substrate (Millipore, Billerica, MA, USA) on X-ray film (CL-Xposure™ film, Thermo Scientific, Rockford, IL, USA).

### Two-step purification method: cationic exchange chromatography and reverse phase-high performance liquid chromatography (RP-HPLC)

A tangential filtration of the conditioned media was previously employed via a Labscale™ TFF System (Merck Millipore, Burlington, MA, USA) with a 5 kDa of molecular weight cutoff to remove any contaminants or reagents that may interfere in the purification. The first purification step consisted of a concentration and purification using an ion exchange chromatographic resin, cationic type, SP-Sepharose Fast Flow (GE Healthcare, Uppsala, Sweden) (Arthuso et al. 2012; Heller et al. 2010; Soares et al. 2006). In this first step, the pH of the conditioned medium of about 7.4 was adjusted to pH 5.0 using glacial acetic acid. The material was then applied to a 13 mm ID × 10 cm column, previously equilibrated with 50 mM sodium acetate (pH 5.0). To remove impurities a wash step was performed with the same buffer plus 90 mM NaCl. Prolactin elution was carried out with 25 mM HEPES buffer, pH 8.0 at a flow rate of 200 mL/h, collecting fractions of 5.0 mL. UV absorbance was evaluated at 220 and 280 nm. The selected fractions were then analyzed by SDS-PAGE and WB. For an efficient separation of G-hPRL, the pooled fractions were loaded onto a Grace-Vydac C4 RP-HPLC column, equilibrated with 0.05 M sodium phosphate buffer containing 45% acetonitrile. The maximum sample volume applied in each RP-HPLC preparative run was 6 mL (3 × 2 mL applied sequentially). G-hPRL was eluted with 50% acetonitrile, pH 7.0, at a flow rate of 0.5 mL/min for 30 min at 30 °C. The pool of the fractions containing G-hPRL was lyophilized for N-glycoprofiling analysis.

### In vitro bioassay

The biological activity of HEK293T-G-hPRL was evaluated by the BaF/3-LLP cell proliferation bioassay (Bole-Feysot et al. 1998; Glezer et al. 2006; Soares et al. 2006). The assay was carried out using the International Standard of Recombinant hPRL of the World Health Organization (WHO 97/714), with a declared potency of 57.2 ± 11.4 IU/mg. Relative potencies were calculated with basis on the ED_50_ of each curve.

### Mass spectrometry for molecular mass determination

The molecular mass determination of recombinant hPRL (NG-hPRL and G-hPRL) was performed via MALDI–TOF-MS by Asparia Glycomics SL, Donostia-San Sebastián, Spain. A diluted glycoprotein sample (1:5, 1:10 and 1:20 from a 1 mg/mL solution) was mixed 1:1 with MALDI matrix solution (sinapinic acid 7 mg/mL in 0.1% trifluoroacetic acid and 50% acetonitrile) and spotted directly to the MALDI plate (1 μL). The analysis was performed in linear positive mode in the range of 5000–40,000 Da in UltrafleXtreme MALDI–TOF-MS equipment (Bruker Daltonics, Bremen, Germany). The Open Source Mass Spectrometry tool data processing software was used for increased resolution analysis in the range 15,000–35,000 Da.

### N-glycoprofiling analysis by permethylation and Matrix-assisted laser desorption ionization time-of-flight mass spectrometry (MALDI–TOF-MS)

All the following procedures and N-glycoprofiling analyses were also carried out at Asparia Glycomics SL, being then interpreted by our research group.

#### Deglycosylation protocol

The glycoprotein sample (35 μL, 1 mg/mL) was denaturated using 4 μL of Glycoprotein Denaturing Buffer (5% SDS, 400 mM DTT, New England Biolabs, Ipsich, MA, USA), heating at 99 °C for 12 min. The denatured protein solution was diluted with 0.4 mL of deionized water and concentrated, using Amicon^®^ Ultra 0.5 mL Centrifugal Filters (10,000 Da molecular weight cutoff) at 12,500 rpm for 3 min at 25 °C, to remove SDS and DTT excess. The retained fraction was diluted again with 0.4 mL of deionized water and concentrated by centrifugation. The concentrated/denatured protein solution was treated with 1.1 μL of PNGase-F solution (CarboClip, Asparia Glycomics) and incubated overnight at 37 °C. After deglycosylation, released N-glycans were isolated from the protein fraction by filtration using the same Amicon Ultra 0.5 mL Centrifugal Filters at 12,500 rpm for 4 min at 25 °C. The filtrate solutions containing released N-glycans were lyophilized in a TELSTAR LyoQuest Plus-55 freeze dryer and the retained fractions were recovered by inverting the filter, transferred by centrifugation at 1500 rpm for 1 min at 25 °C into a receiver vial and stored at − 20 °C.

#### Permethylation of released N-glycans

The glycan sample dried down in the glass tube was re-dissolved in a slurry of finely ground NaOH pellets in dimethyl sulfoxide (75 μL), followed by the addition of 35 μL of methyl-iodide. The reaction mixture was gently vortexed and incubated for 2 h at 4 °C and then quenched and neutralized on ice with 0.2 mL H_2_O and 0.15 mL 30% acetic acid, reaching pH 6. The solution was then applied directly to a pre-washed and equilibrated C18 Sep-Pak cartridge (Waters, Milford, MA, USA). Permethylated glycans were collected in the 50% acetonitrile fraction, as well as in the later eluting 75–100% acetonitrile fraction containing the larger ones.

#### MALDI–TOF-MS analysis of N-glycans

The fractionated sample containing permethylated N-glycans from human glycosylated prolactin was evaporated to dryness before analysis. The dried sample was reconstituted in 10 μL deionized water and loaded directly to the MALDI plate (1 μL). DHB matrix (20 mg/mL in acetonitrile) was used and the sample analyzed in reflector positive mode in the 800–4500 Da mass range. N-Glycan structure assignment was performed using the Expasy Glycomod tool (https://web.expasy.org/glycomod). The parameters for structure assignation were: (a) mass tolerance: ± 0.8 Da; (b) ion mode and adducts: Na^+^; (c) form of N-linked oligosaccharide: free/PNGase F released oligosaccharides; (d) monosaccharide residues: permethylated.

### Average N-glycan mass and monosaccharide molar ratio determination on the basis of glycoprofiling analysis

The N-glycoprofilings and the relative percent intensity of each determined glycan were used to calculate the average N-glycan mass that is present in the HEK-G-hPRL molecule. Through this stoichiometric approach the contribution of each monosaccharide to each glycan was also determined. All calculations have been carried out as detailed in previous work and related additional data (Capone et al. 2015).

## Results

**Fig. 1:**
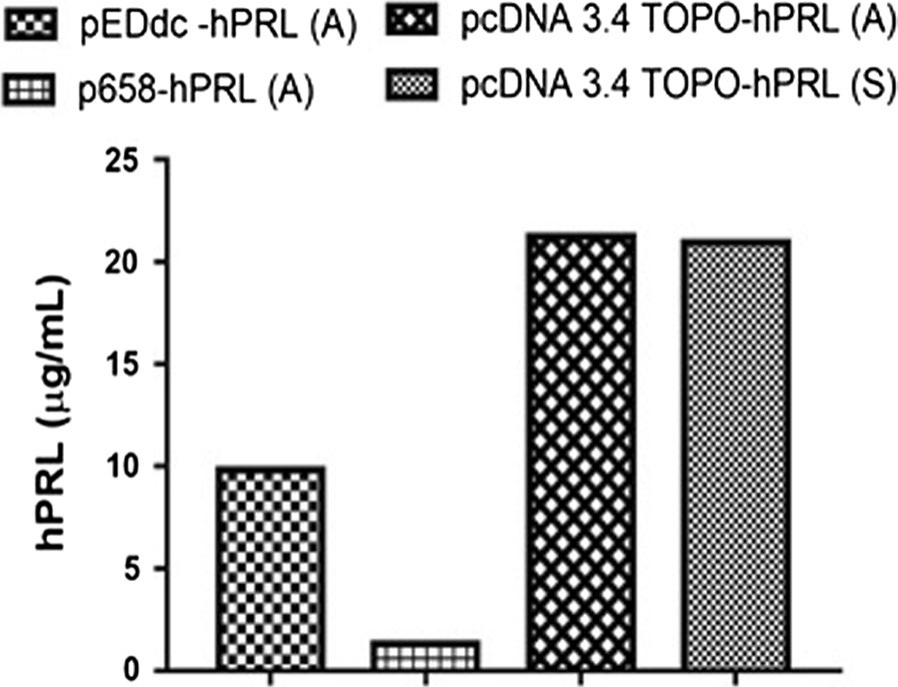
Expression levels of hPRL by RP-HPLC analysis of conditioned media collected for 4 days from HEK293T adherent (A) and from HEK293F suspension (S) cells, transiently transfected with different vectors (pEDdc-hPRL, p658-hPRL, pcDNA 3.4 TOPO-hPRL)

As we can observe in Fig. 1, the most efficient vector was pcDNA 3.4 TOPO^®^, which provided ~ 20 mg/L of the hormone in either adherent or suspension cells.

**Fig. 2:**
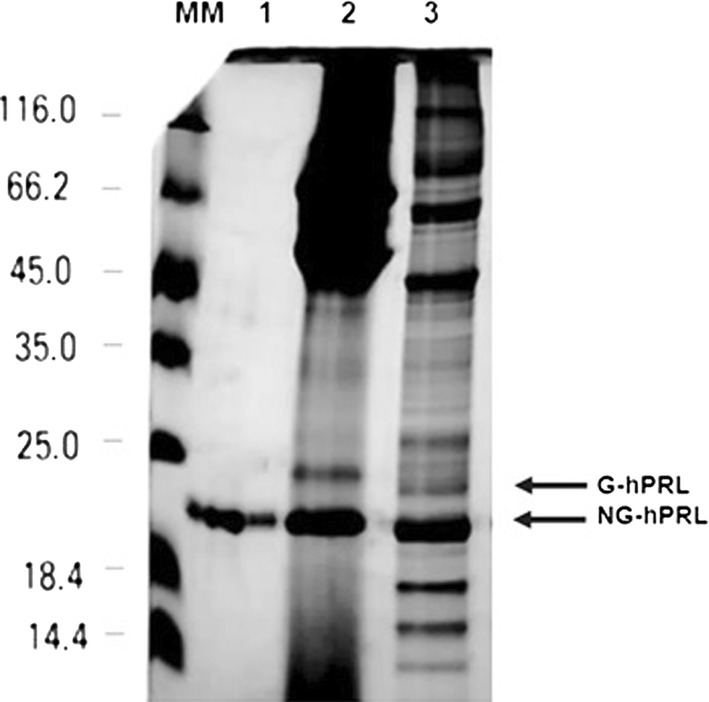
SDS-PAGE analysis comparing hPRL from conditioned media collected from HEK293T adherent cells with that from HEK293F suspension cells, all transiently transfected with the pcDNA 3.4 TOPO-hPRL vector, after SP-Sepharose FF purification step. MM, molecular mass marker; (1) internal reference preparation of NG-hPRL from *E. coli*; (2) hPRL obtained from HEK293T adherent cells; (3) hPRL obtained from HEK293F suspension cells

**Fig. 3:**
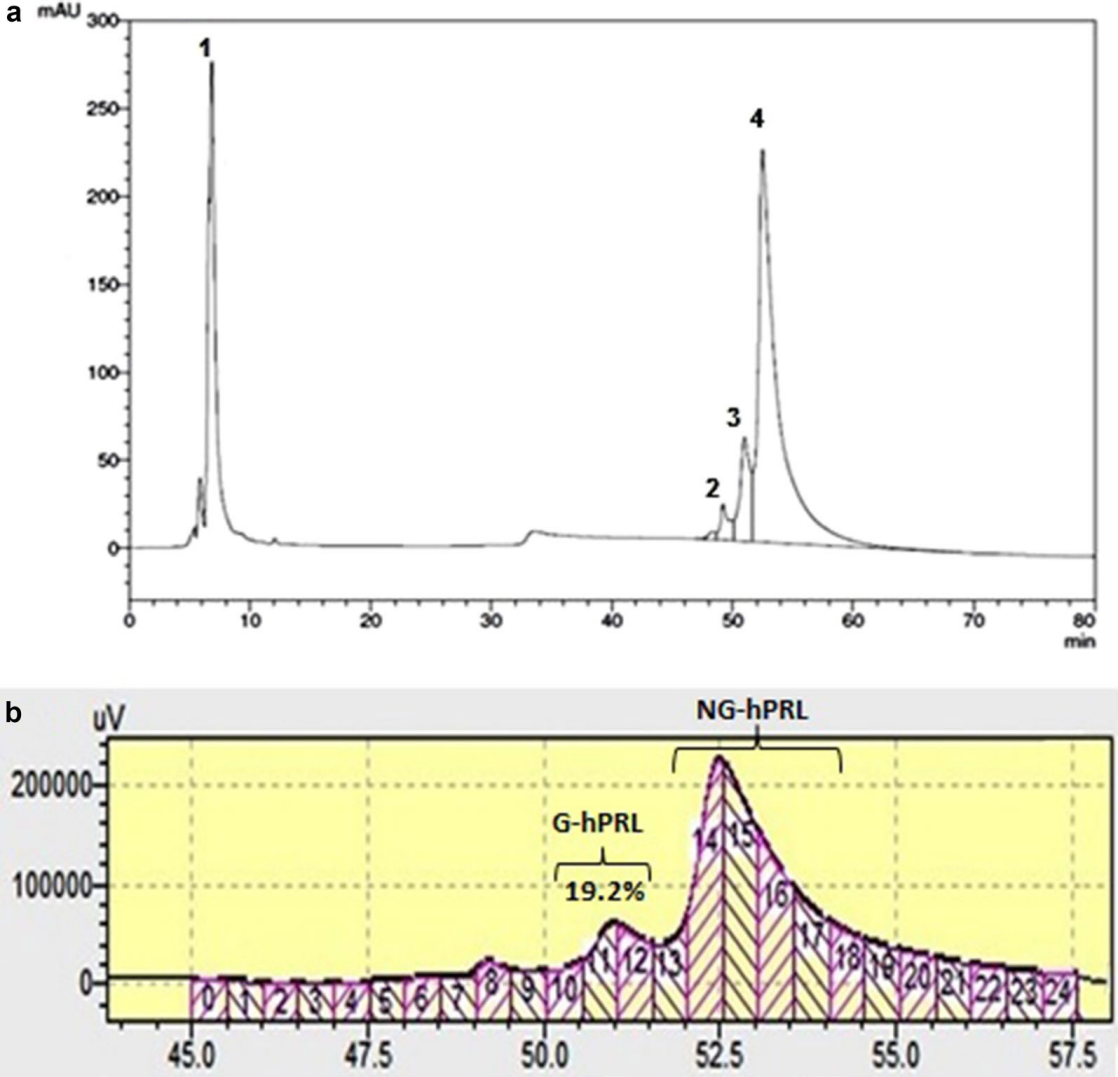
Separation of G-hPRL from NG-hPRL via RP-HPLC: **a** chromatogram showing the presence of four peaks: (1) eluted material without hPRL; (2) small fraction possibly due to a carbohydrate heterogeneity of HEK-G-hPRL; (3) main peak of HEK-G-hPRL; (4) NG-hPRL. **b** The same RP-HPLC chromatographic step, here expanded to show how the three fractions (#10–11–12) were collected for N-glycoprofiling determination

Considering expression efficiency and the higher yields obtained, our studies were carried out just utilizing pcDNA 3.4 TOPO-hPRL vector in transiently transfected HEK293F suspension cells. About 19% of the prolactin obtained was glycosylated (G-hPRL) as shown in Fig. 2 and was separated from non-glycosylated prolactin (NG-hPRL) via a RP-HPLC methodology already set up in previous work (Capone et al. 2015), as presented in Fig. 3. Fractions #10, 11, and 12 were collected as shown in Fig. 3b and used for N-glycoprofiling determination.

The percent of glycosylated fraction (19.2% on total prolactin) is in agreement with previous literature values, considering that purified pituitary-derived prolactin has been reported to contain 5–30% of glycosylated form while, in our hands, CHO-derived prolactin has shown the presence of ~ 10% of G-hPRL (Heller et al. 2010; Price et al. 1995; Soares et al. 2000). In addition, purification using RP-HPLC (Fig. 3) evidenced a third peak, probably due to a carbohydrate heterogeneity of HEK-G-hPRL as already reported for native and CHO-derived G-hPRL (Capone et al. 2015; Haro et al. 1990).

**Fig. 4:**
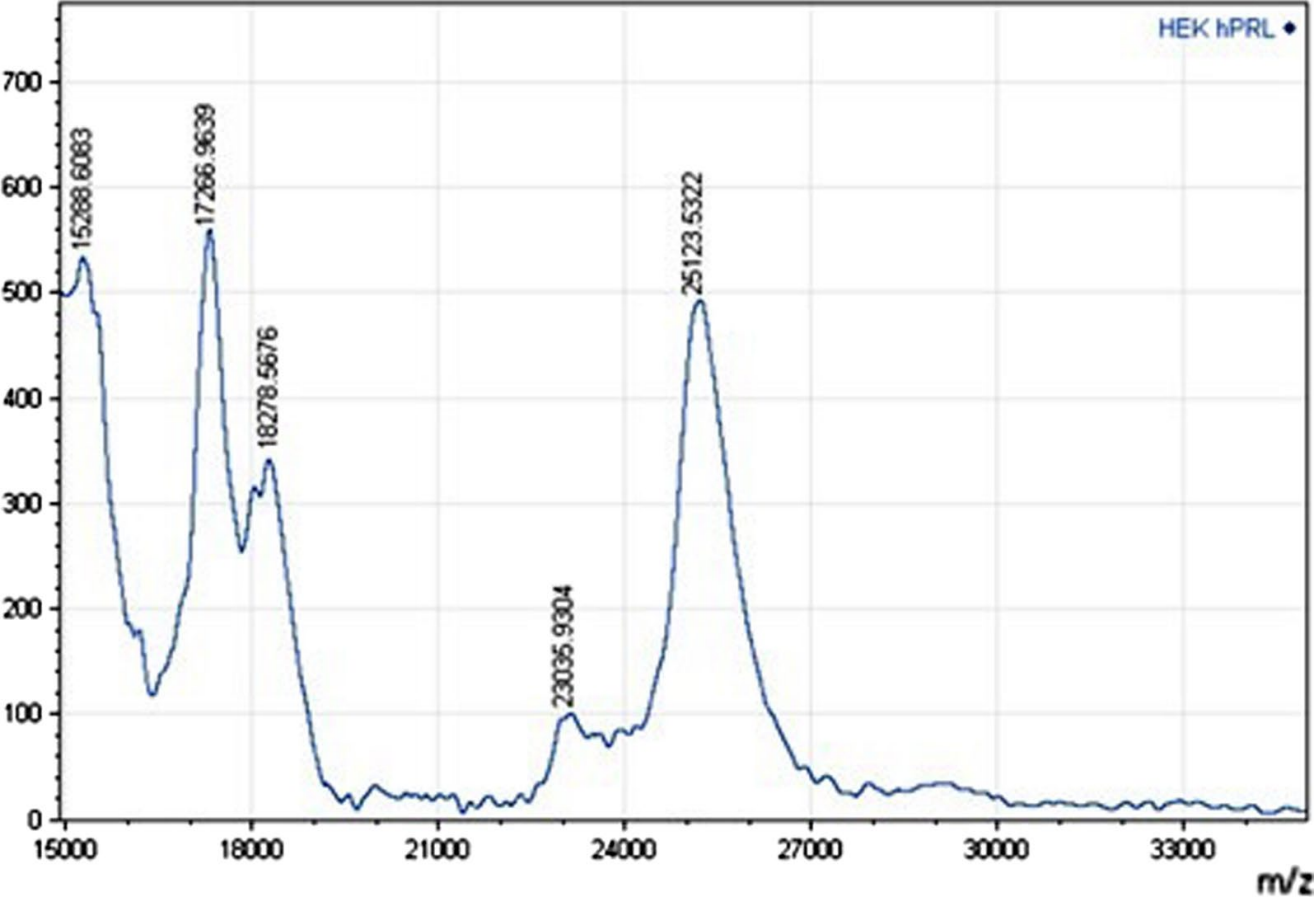
HEK293-G-hPRL molecular mass determination by MALDI–TOF-MS

The relative molecular mass (Mr) determination by MALDI–TOF-MS is shown in Fig. 4, where we can appreciate, in the same experiment, the mass of HEK-G-hPRL and of a small amount of HEK-NG-hPRL.

Concerning the in vitro bioactivity of CHO-derived hPRL variants, we emphasize, as mentioned, that G-hPRL bioactivity is ca. fourfold lower than that of NG-hPRL (~ 64 IU/mg) and among G-hPRL of different origins, we can cite 15–20 IU/mg for CHO-derived and only 3.6 IU/mg for pituitary-derived G-hPRL (Capone et al. 2015; Heller et al. 2010; Rafferty et al. 2001).

**Fig. 5:**
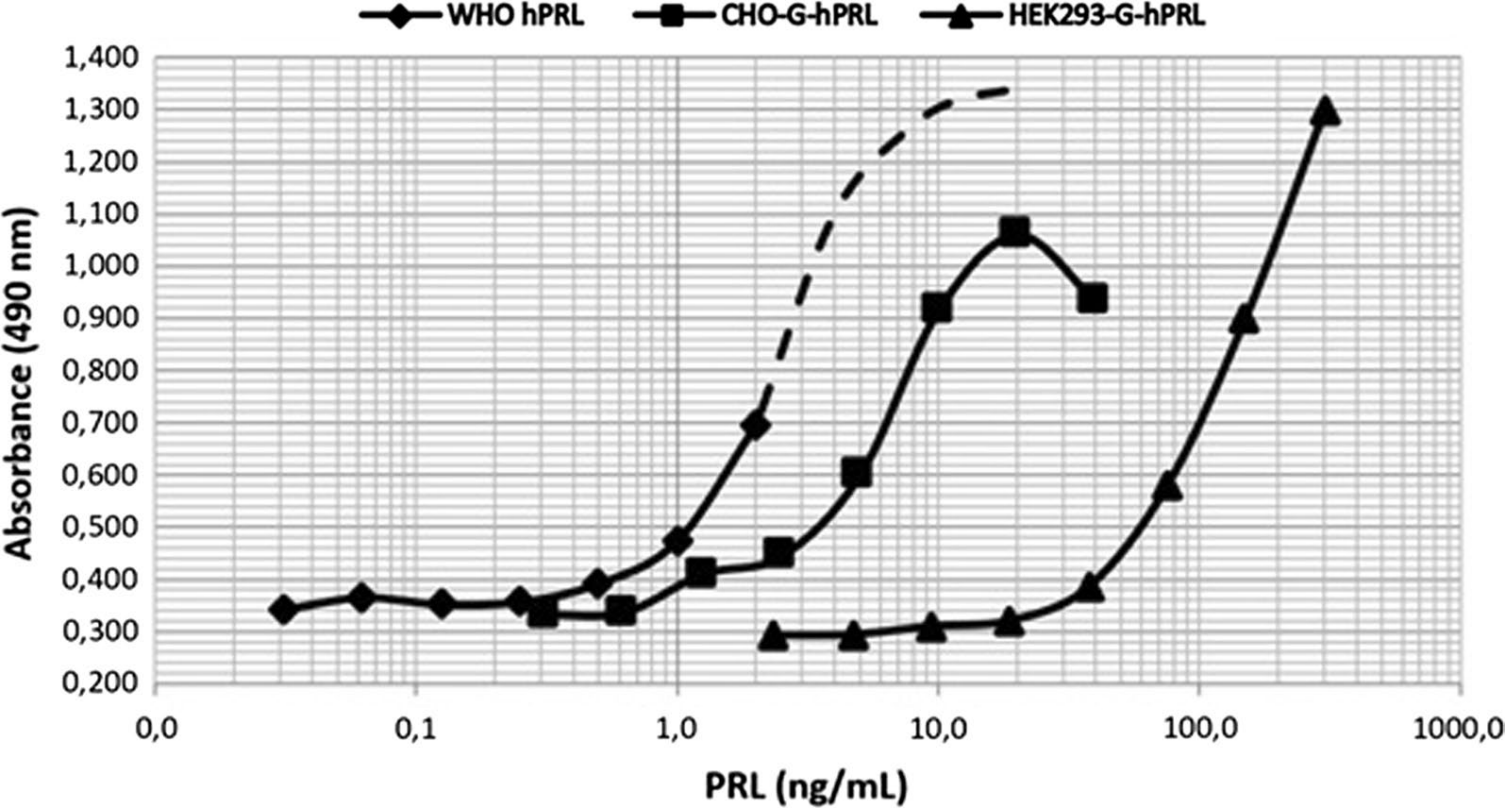
Bioactivity determination of G-hPRL of different origins via the BaF/3-LLP in vitro bioassay: relative potencies have been determined by comparing the ED_50_ of the different curves and considering the nominal activity of 57.2 IU/mg for the International Standard of hPRL (WHO 97/714)

In the present study CHO-G-hPRL confirmed a potency of 24.6 ± 0.54 IU/mg, while HEK-G-hPRL showed only of 0.92 ± 0.02 IU/mg, making of this form the one with the lowest biological activity we ever determined (Fig. 5). The biological potencies were determined according to the related ED_50_ values: 2.6 ng/mL, 6.0 ng/mL and 160 ng/mL for WHO International Standard of hPRL (97/714, 57.2 IU/mg), CHO-G-hPRL and HEK-G-hPRL, respectively, which provided relative activities of 0.43 and 0.016 for CHO-G-hPRL and HEK-G-hPRL, respectively.

**Fig. 6:**
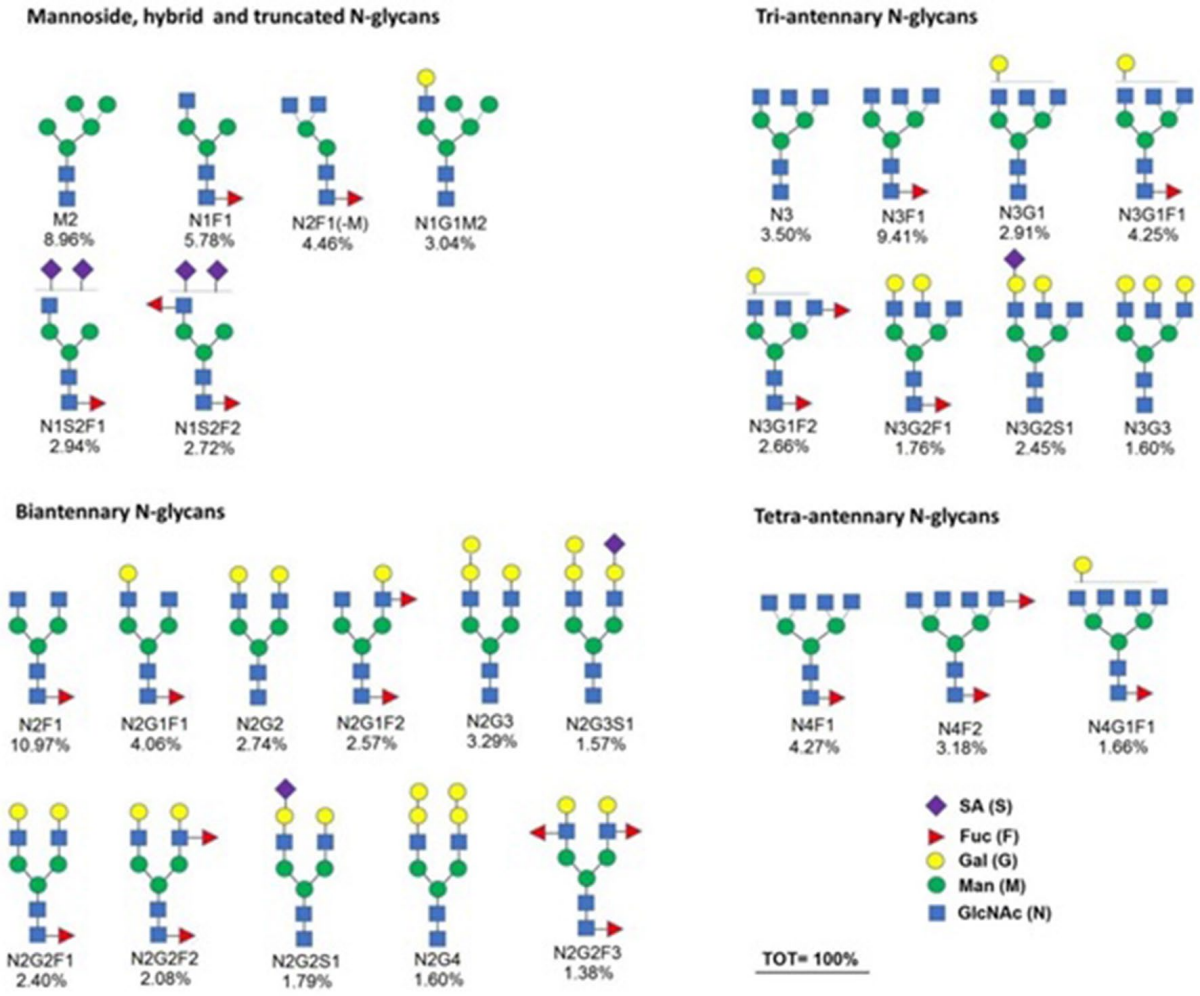
N-glycan structures of HEK-G-hPRL: the relative percent intensity is indicated below each glycan structure

The 28 different N-glycan structures identified in the HEK-G-hPRL carbohydrate moiety via N-glycoprofiling, are shown in Fig. 6, together with the relative percent intensity, while Table 1 reports the underivatized mass of each specific N-glycan and its intensity in comparison with those identified in previous work and concerning CHO-G-hPRL and pit-G-hPRL (Capone et al. 2015).

**Table 1:** Different N-glycan structures and relative intensities for the native and two recombinant preparations of G-hPRL

	N-Glycan	Underivatized mass (–H_2_O) (Da)	Relative intensity of each N-glycan per each preparation (%)		
			HEK-G-hPRL	CHO-G-hPRL	Pit-G-hPRL
1	0	892.3	–	–	3.9
2	F1	1038.4	–	–	8.2
3	M1	1054.4	–	–	0.4
4	M2	1216.4	9.0	4.6	0.7
5	N1F1	1241.5	5.8	–	0.3
6	N2F1(-M)	1282.5	4.5	–	–
7	M2P1	1296.4	–	–	1.1
8	M3	1378.5	–	1.0	0.5
9	N1G1F1	1403.5	–	–	0.4
10	N2F1	1444.5	11.0	–	0.5
11	M3P1	1458.5	–	7.6	3.1
12	N3	1501.6	3.5	–	–
13	N1G1M2	1581.6	3.0	–	–
14	N2F2	1590.6	–	–	0.5
15	N2G1F1	1606.6	4.1	1.3	0.5
16	N2G2	1622.6	2.7	–	–
17	N3F1/N2Gn1F1	1647.6	9.4	–	0.6
18	N3G1	1663.6	2.9	–	–
19	M1N1Gn1F1(SO_4_)1	1686.6	–	–	3.2
20	N1G1S1F1	1694.6	–	0.9	0.6
21	M1N1G1S1	1710.6	–	1.1	–
22	N2Gn1F1(SO_4_)1	1727.6	–	–	1.8
23	N2G1F2	1752.7	2.6	–	0.8
24	N2G2F1	1768.6	2.4	5.0	–
25	N2G3	1784.6	3.3	–	–
26	N2Gn1F2	1793.7	–	–	1.4
27	N3G1F1	1809.7	4.2	–	–
28	N1S2F1	1823.6	2.9	–	–
29	N4F1	1850.7	4.3	–	–
30	N2G1Gn1F1(SO_4_)1	1889.6	–	–	1.3
31	N2G1S1F1	1897.7	–	0.6	–
32	N2G2S1	1913,7	1.8	1.8	–
33	N2G2F2	1914.7	2.1	–	0.5
34	N2Gn2F1(SO_4_)1	1930.6	–	–	4.1
35	N2G4	1946.7	1.6	–	–
36	N3G1F2	1955.7	2.7	–	–
37	N1S2F2	1969.7	2.7	–	–
38	N3G2F1	1971.7	1.8	–	–
39	N3G3	1987.7	1.6	–	–
40	N4F2	1996.7	3.2	–	–
41	N2Gn2F2	1996.8	–	–	0.6
42	N2Gn2F1(SO_4_)2	2010.6	–	–	28.1
43	N4G1F1	2012.8	1.7	–	–
44	N2G1Gn1F2(SO_4_)1	2035.7	–	–	1.7
45	N2G2S1F1	2059.7	–	12.0	0.5
46	N2G2F3	2060.8	1.4	–	–
47	N2G3S1	2075.7	1.6	–	–
48	N2Gn2F2(SO_4_)1	2076.7	–	–	33.8
49	N3G2S1	2116.8	2.4	–	–
50	N2Gn2F3	2142.8	–	–	0.5
51	N2G2S2	2204.8	–	6.9	–
52	N2G2S2F1	2350.8	–	56.1	0.6
53	N3G3S2F1	2715,9	–	0.8	–
54	N3G3S3	2861.0	–	0.3	–
	*Number of N-glycan structures*		*n = 28*	*n = 14*	*n = 28*
	*Fucosylated glycans (%)*		*66.8*	*76.7*	*90.5*
	*Sialylated glycans (%)*		*11.4*	*80.5*	*1.7*
	*Sulfated glycans (%)*		–	–	*74.0*

Abbreviations for N-glycans were made by not considering the basic pentasaccharide nucleus (“zero”) and adding all other monosaccharides, as stated in Table 1 and Fig. 6, in the following order: Man (M); GlcNAc (N); Gal (G); GalNAc (Gn); NeuAc/sialic acid (S); Fuc (F). So, for example, NeuAc1 Gal1 GlcNAc2 Fuc1 + Man3 GlcNAc2, becomes N2G1S1F1 (Capone et al. 2015)

From Table 1 we can also confirm that the percentage of sialylated glycans (11.4%) even being quite higher than it was for pit-G-hPRL (1.7%) or even for HEK-hTSH (4.7%) (Sant’Ana et al. 2018), still is much lower when compared to that found in CHO-derived G-hPRL (80.5%). The percentage of fucosylated glycans was found quite high in native and recombinant G-hPRL (> 60%), especially in comparison with native and CHO-derived hTSH, whose percent of fucosylated glycans was 35.2 and 11.9 respectively (Ribela et al. 2017).

In Table 2 one can compare the molecular masses of HEK-G-hPRL and the mass fraction due to the carbohydrate moiety, as determined via N-glycoprofiling (MM) and via MALDI–TOF-MS (Mr). In Table 3 we can observe a comparison between the molecular masses of G-hPRL of different origins as determined via the above mentioned two different methods, while in Table 4 the monosaccharide contribution to HEK-derived G-hPRL is reported as calculated exclusively from N-glycoprofilings.

**Table 2:** Molecular mass of HEK cell-derived glycosylated prolactin obtained via N-glycoprofiling analysis and compared to MALDI–TOF-MS determination

Via N-glycoprofiling			Via MALDI–TOF-MS			
Average N-glycan mass (Da)	G-hPRL MM (Da)^a^	Carbohydrate moiety (%)^b^	NG-hPRL (Mr)	G-hPRL (Mr)	Carbohydrate moiety (%)	Difference between G-hPRL MM and Mr (%)
1656.3	24,554.1	6.7	23,035.9	25,123.5	8.3	− 2.27

^a^Calculated by adding the average N-glycan mass to the calculated NG-hPRL mass of 22,897.75 (Capone et al. 2015)

^b^Calculated as a percent of the average glycan mass on G-hPRL MM

**Table 3:** Comparisons between the molecular masses of G-hPRL of different origins, determined by MALDI–TOF-MS (Mr) and by N-glycoprofiling (MM)

Host cell	MALDI–TOF-MS (Da)	N-Glycoprofilings (Da)	Difference MM/Mr (%)
Human lactotrophs^a^	24,903	24,736	− 0.68
CHO cells^a^	24,970	25,016	+ 0.18
C127 cells^a^	25,139	–	–
HEK293 cells^b^	25,124	24,554	− 2.27

^a^From Capone et al. (2015)

^b^From the present work

**Table 4:** Monosaccharide/HEK-G-hPRL molar ratio determination based on N-glycoprofiling

	Fraction of glycan mass (%)	Monosaccharide weight contribution (Da)	Mole/G-hPRL mole
Fuc	7.20	119.3	0.82
GlcNAc	50.98	844.4	4.16
Gal	6.71	111.1	0.69
Man	32.77	542.8	3.35
SA	2.55	42.2	0.14

Considering the average N-glycan mass = 1656.3 Da

## Discussion

Human recombinant prolactin has been synthesized for the first time in a human host: HEK293 cells, either adherent (A) or in suspension (S) culture, using three different expression vectors. The pcDNA 3.4 TOPO-PRL expression vector was clearly the most efficient and, for practical reasons, it was used in transiently transfected suspension cell culture for the subsequent studies. The two variants obtained (NG-hPRL and G-hPRL) were then purified and characterized by SDS-PAGE, RP-HPLC and, in particular, HEK-derived G-hPRL also by in vitro bioassay. Considering the declared bioactivity of the WHO International Standard of hPRL 97/714 (57.2 IU/mg), the bioactivity of CHO-G-hPRL and of HEK-G-hPRL was determined as 24.6 and 0.92 IU/mg respectively, the latter being the lowest we ever found, i.e. ~ fourfold lower than that determined in previous work for native G-hPRL (Capone et al. 2015).

The main goal of our study, however, was the analysis of the N-glycan structures present in HEK-G-hPRL, considering that in previous work we already compared in this respect, pituitary-derived G-hPRL with CHO-derived G-hPRL (Capone et al. 2015), pituitary-derived hTSH with CHO-derived hTSH (Ribela et al. 2017) and these same products with HEK-derived hTSH (Sant’Ana et al. 2018). We soon observed that human cells, either from native or from embryonic kidney cells, produced a much higher number of different N-glycan structures (n = 28), while CHO cells only produced 14, a number practically matching with that obtained analyzing CHO-hTSH (n = 15). Only 5 structures of G-hPRL, out of a total of 54, were found in common between the two human hosts, while there are only 4 structures in common between the two recombinant preparations. Forty structures are unique in the three preparations. More interestingly: there are 15 structures in common between HEK-G-hPRL and HEK-hTSH confirming, as already observed, that mostly the host and not the synthesized protein sequence is influencing the N-glycan type (Ribela et al. 2017).

The molecular masses of HEK-NG-hPRL and HEK-G-hPRL determined by MALDI–TOF-MS are both in agreement with previously reported literature values and could define an average N-glycan mass of 2087.6 Da, which is relatively different from that calculated directly from N-glycoprofiling: 1656.3 Da. Since in previous work we always obtained a better agreement between the two types of calculations either for G-hPRL or for hTSH, we could speculate that a fragmentation of the analyzed N-glycans may have occurred or even that the NG-hPRL mass determined in the experiment reported in Fig. 4, where it is present in a very small amount, might not have been accurate enough. The latter hypothesis, however, would not be supported by the fact that the HEK-NG-hPRL mass of 23,036 Da is perfectly acceptable considering the previously mentioned inter-assay, inter-preparation statistics which defined an average value of 22,910.3 ± 32.22 Da (CV = 0.14%, n = 7) for hPRL non-glycosylated form (Capone et al. 2015).

Concerning the monosaccharide contribution to HEK-derived G-hPRL, reported in Table 4, we can observe the very low mole per mole number of sialic acid (0.14), emphasizing that in native, pit-G-hPRL it was even lower (0.02) (Capone et al. 2015). It is of note that the SA/Gal molar ratio, whose constant value of 0.60–0.80 has been reported for hTSH, either of native or of CHO origin and also for CHO-derived G-hPRL, fell down to 0.20–0.25 in the case of pituitary and HEK-derived G-hPRL (Capone et al. 2015; Ribela et al. 2017).

Considering that our main interest is related to glycoprotein hormones, we did not focus other important recombinant protein synthesis still based on CHO or HEK293 cell culture. We just recall some important works based on human cell lines in general (Fliedl et al. 2015; Mortazavi et al. 2019; Zhong et al. 2019), on CHO cell (Bohm et al. 2015; Croset et al. 2012; Durocher and Butler 2009; Zhang et al. 2010) and on HEK293 (Bohm et al. 2015; Bouvette et al. 2018; Croset et al. 2012; Ding et al. 2017; Gugliotta et al. 2017; Hu et al. 2018; Swiech et al. 2012; Zhang et al. 2010).

In conclusion, for the first time recombinant human prolactin, a hormone largely applied in human diagnostics, has been synthesized in human embryo kidney cells, showing to contain ~ 19% of the glycosylated form (G-hPRL). Even considering that the physiological significance of this glycosylated variant of the hormone is still not well elucidated, it represents a particularly interesting model of simple glycoprotein, containing only one potential N-glycosylation site, never completely occupied. Being HEK cells of human origin, the recombinant hormone here obtained presented some similarities with the natural form of G-hPRL, which were not found in the CHO-derived product, i.e. similar number of N-glycan structures, very low sialylation level and also very low biological activity. Our main objective has always been the comparison between the recombinant and natural structures of glycohormones that are largely applied in human diagnosis and therapy, applications that frequently do not consider the existence of fundamental differences, mostly determined by the host cells in which these biopharmaceuticals are synthesized.

## Data Availability

Not applicable.
